# Inflammation From Peripheral Organs to the Brain: How Does Systemic Inflammation Cause Neuroinflammation?

**DOI:** 10.3389/fnagi.2022.903455

**Published:** 2022-06-16

**Authors:** Yuanjie Sun, Yoshihisa Koyama, Shoichi Shimada

**Affiliations:** ^1^Department of Neuroscience and Cell Biology, Osaka University Graduate School of Medicine, Osaka, Japan; ^2^Addiction Research Unit, Osaka Psychiatric Research Center, Osaka Psychiatric Medical Center, Osaka, Japan

**Keywords:** neuroinflammation, systemic inflammation, organ-brain axis, peripheral organs, cytokines, microcyte

## Abstract

As inflammation in the brain contributes to several neurological and psychiatric diseases, the cause of neuroinflammation is being widely studied. The causes of neuroinflammation can be roughly divided into the following domains: viral infection, autoimmune disease, inflammation from peripheral organs, mental stress, metabolic disorders, and lifestyle. In particular, the effects of neuroinflammation caused by inflammation of peripheral organs have yet unclear mechanisms. Many diseases, such as gastrointestinal inflammation, chronic obstructive pulmonary disease, rheumatoid arthritis, dermatitis, chronic fatigue syndrome, or myalgic encephalomyelitis (CFS/ME), trigger neuroinflammation through several pathways. The mechanisms of action for peripheral inflammation-induced neuroinflammation include disruption of the blood-brain barrier, activation of glial cells associated with systemic immune activation, and effects on autonomic nerves via the organ-brain axis. In this review, we consider previous studies on the relationship between systemic inflammation and neuroinflammation, focusing on the brain regions susceptible to inflammation.

## Introduction

Inflammation in the brain has drawn widespread attention due to its implication in several diseases at multiple stages of life. For instance, some studies have suggested a relationship between neuroinflammation and several types of dementia (Bevan-Jones et al., 2020). Maternal infection is shown to cause neuroinflammation in the fetal brain, leading to schizophrenia (Brown et al., 2004) and neurodevelopmental issues such as autism spectrum disorder (ASD) (Slawinski et al., 2018). In addition, research on major depressive disorder (MDD) revealed that C-reactive protein (CRP) could serve as a marker of neuroinflammation and peripheral inflammation and is well-suited for guiding immunotherapy targeting inflammatory cytokines such as TNF-α and IL-6 in the patient with MDD (Felger et al., 2020). Therefore, neuroinflammation significantly affects psychological symptoms, and we need to control neuroinflammation.

Several insults can cause neuroinflammation, such as viral infection in the central nervous system (Klein et al., 2019), peripheral inflammation [chronic joint pain (Schrepf et al., 2018), gut inflammation (Do and Woo, 2018)], and autoimmune issues. For autoimmune issues, a famous example would be paraneoplastic limbic encephalitis (PLE). In patients with specific types of cancer, such as small cell lung cancer, the immune system cross-reacts with distinct onconeural antigens would cause a damage to neural tissue and trigger neuroinflammation (Bien et al., 2012; Zhang et al., 2013). Taken together, persistent systemic inflammation increases the likelihood of neuroinflammation. Meanwhile, factors such as lifestyle also contribute to neuroinflammation. Unhealthy eating habits have been shown to influence the balance of intestinal microbiota, change the blood-brain barrier (BBB) permeability, and cause neuroinflammation (Dutheil et al., 2016; Spielman et al., 2018). Obesity, metabolic syndrome, and diabetes can accelerate the metabolism of neuronal cells, which produced reactive oxygen species (ROS) causing oxidative stress and consequently neuroinflammation (de Heredia et al., 2012; Van Dyken and Lacoste, 2018). Moreover, mental stress has been shown to increase the levels of several cytokines, such as tumor necrosis factor-alpha (TNF-α) and interleukin-1 (IL-1) to trigger neuroinflammation and induce depression or anxiety. Sleep loss is also suggested to be a potential cause of neuroinflammation (Curcio et al., 2006; Hurtado-Alvarado et al., 2016). Thus, anyone can be at risk of neuroinflammation, with or without pathology. Therefore, it is essential to elucidate the mechanism of neuroinflammation onset.

Among the known causes of neuroinflammation, the influence of systemic inflammation on neuroinflammation has scarcely been explored. Recent evidence suggests that chronic peripheral inflammation causes systemic inflammation which may enhance the synthesis of pro-inflammatory cytokines and other inflammation-promoting mediators, activating neuroinflammation in the diseased brain (Perry, 2004; Aktas et al., 2007). Some studies have pointed out the relationship between systemic inflammation and microglial activation via multiple neurotoxic factors, including TNF-α, IL-1, and ROS (Hoogland et al., 2015). Microglia activation is the principal driver of inflammation in the brain. It has been suggested that chronic inflammation breaks down the BBB, degrading the separation of central and peripheral circulation system, leaving the central nerves system (CNS) vulnerable (Abbott et al., 2010). The close bidirectional relationship of the gut-brain axis, which includes neural, hormonal, and immune communication also plays a vital role in neuroinflammation caused by systemic inflammation (Abautret-Daly et al., 2018). Thus, the persistence of peripheral inflammation causes systemic inflammation and the enhancement of pro-inflammatory factors and disruption of the brain tissue protection, all lead to neuroinflammation.

Another interesting point is that the lesion sites are often limited in the case of neuroinflammation caused by systemic inflammation. Taking PLE as an example, though antigens are expressed throughout the brain, neuroinflammation is limited to the limbic system (Bien et al., 2012; Zhang et al., 2013). In addition, the previous study also pointed out that inflammatory bowel disease (IBD) could lead to neuroinflammation in a bottom-up manner, and this effect depended on the brain region: the upregulation of cyclooxygenase-2 (COX-2) mRNA, glial fibrillary acidic protein (GFAP) mRNA, and GFAP expression during exposure to dextran sulfate sodium salt (DSS) in the hippocampus; upregulation of COX-2 mRNA only 3 days after DSS treatment in the hypothalamus; downregulation of brain-derived neurotrophic factor (BDNF) and COX-2 mRNAs in the amygdala (Do and Woo, 2018). Among those previous studies, the affected regions in the brain are limited such as the cortex, striatum, thalamus, hippocampus, the amygdala. Therefore, it is essential to clarify the differences in pathophysiology of neuroinflammation in the different affected regions.

Having a deep understanding of the mechanism does favor the development of new medicines for diseases caused by neuroinflammation. A meta-analysis suggested that anti-inflammatory add-on treatment affects psychotic disorders, though no superiority was found in primarily anti-inflammatory medicines (Jeppesen et al., 2020). After administration of anti-inflammatory medicines, they mainly stay in the peripheral blood circulation. Plasma protein binding limits brain Non-Steroidal Anti-Inflammatory Drug (NSAID) uptake by reducing the free fraction of NSAID in the circulation. As only the unbinding small molecular could pass through the BBB, it is extremely difficult for therapeutics molecules enter the CNS (Rhea and Banks, 2019). As a result, even though the dose of an anti-inflammatory medicine administered via intraperitoneal or intravenous injection is significantly increased, few anti-inflammatory substances enter the brain. Given the safety of medicine administration, dose beyond the permissible range of the peripheral immune system is not recommended. To deal with this problem, a research team has recently developed a new route of administration for drugs - trans spinal delivery by transdermal patch over the neck and cervical spine (Lehrer and Rheinstein, 2019). The medicines prescribed by the above-mentioned administration method diffuse through the intervertebral spaces and enter the cerebrospinal fluid, which is not influenced by BBB.

In this review, we summarize previous studies on the relationship between systemic inflammation and neuroinflammation in detail, focusing on the brain regions susceptible to inflammation. This discussion could be an overview of the neuroinflammation induced by systematic inflammation. By figuring out essential factors or pathway in this process, we could provide ideas for further research on this topic. If the mechanism could be discovered in the future, it will also be easier to prevent the occurrence of neuroinflammation, reduce the suffering of patients, medical expenses, and social burden.

## Previous Studies on the Influence of Systemic Inflammation on the Brain

Previous studies have shown that inflammation in several peripheral organs leads to inflammation in the brain. Colitis may be the most discussed disease that causes neuroinflammation. In the colitis animal model (Abautret-Daly et al., 2018; Ticinesi et al., 2018; Peppas et al., 2021; Craig et al., 2022), neuropsychiatric symptoms such as impaired spatial and recognition memory (Peppas et al., 2021), depression (Riazi et al., 2015; Abautret-Daly et al., 2018; Do and Woo, 2018; Peppas et al., 2021), cognitive dysfunction (Riazi et al., 2015; Do and Woo, 2018; Ticinesi et al., 2018), altered stress-associated behavior (Reichmann et al., 2015), and anxiety (Riazi et al., 2015; Abautret-Daly et al., 2018; Peppas et al., 2021) are observed in behavioral experiments. The affected regions in the brain are the hippocampus (Reichmann et al., 2015; Riazi et al., 2015; Zonis et al., 2015; Do and Woo, 2018; Han et al., 2018; Han et al., 2020; Peppas et al., 2021), cortex (Han et al., 2018; Peppas et al., 2021), amygdala (Reichmann et al., 2015; Do and Woo, 2018), and the hypothalamus (Reichmann et al., 2015; Do and Woo, 2018). Imaging studies in patients also corroborate this relationship between colitis and brain lesions (Table 1) (Dolapcioglu and Dolapcioglu, 2015).

**TABLE 1:** Animal experiments on the relationship between colitis and inflammation in the brain.

Research	Disease model & Method	Animal	Neuropsychiatric symptoms	Affected regions
He et al. (2021)	1% (wt./vol) DSS	C57BL/6J, NLRP3 knockout (KO) &WT mice (M and F 16-month-old)	Impaired spatial and recognition memory	cortexhippocampus
Do and Woo (2018)	3% (wt./vol) DSS for 3 or 7 days.	C57BL/6J mice (Male, 7-8 week)	Depression, cognitive dysfunction	hippocampus amygdalahypothalamus
Reichmann et al. (2015)	2% (wt./vol) DSS for 7 days	C57BL/6N mice (Male, 10 week)	Altered stress-associated behavior	hippocampus amygdalahypothalamus
Han et al. (2018)	5% (wt./vol) DSS for 7 days	C57BL/6 mice (Male, 7–8 week)	No	cortexhippocampus
Zonis et al. (2015)	3%(wt./vol) DSS for 7 days	C57Bl/6 (Female, 8 week)	Not mentioned	hippocampus
Han et al. (2020)	3% DSS for five days with exposure to hypoxic conditions for 2 days	C57BL/6 mice(Male, 7-8 week)	not mentioned	hippocampus
Riazi et al. (2015)	Intracolonic administration of TNBS (0.5 ml, 50 mg/ml, in 50% ethanol/saline mixture)	Sprague Dawley rats (Adult male)	anxiety, depression, and cognitive dysfunction	hippocampus

*Dss, dextran sodium sulfate; TNBS, 2,4,6-trinitrobenzenesulfonic acid.*

Other animal models have been used to illustrate the relationship between inflammatory diseases and neuroinflammation, such as periodontal disease (PD), gastritis, pancreatitis, arthritis, cystitis, and atopic dermatitis (Lin et al., 2018; Albaret et al., 2020; Ding et al., 2020; Furutama et al., 2020; Matsushita et al., 2021; Shin et al., 2021). The inflammation regions in the brain and neuropsychiatric symptoms for these studies are listed in Table 2. In addition, some clinical experiments also provided preliminary evidence that primary biliary cholangitis (PBC) (Zenouzi et al., 2018), chronic fatigue syndrome/myalgia encephalomyelitis (CFS/ME) (Nakatomi et al., 2014), and chronic obstructive pulmonary diseases (COPD) (Pelgrim et al., 2019) have some relationship with neuroinflammation.

**TABLE 2:** Animal experiments on the relationship between inflammation in peripheral organs and in the brain.

Research	Disease model and Mehod	Animal	Neuropsychiatric symptoms	Affected regions
Furutama et al. (2020)	Periodontal disease (PD)	C57BL/6J mice (8- to 12-week-old females)	Neurodegenerative disorder	Hippocampus
Albaret et al. (2020)	Gastritis	C57BL/6 mice (6-week-old females)	Alzheimer’s disease (AD)	Not mentioned
Lin et al. (2018)	pancreatitis	Adult male Sprague Dawley rats	Not mentioned	Not mentioned
Matsushita et al. (2021)	Arthritis	DBA/1J mice (Male)	Fatigue, depression, hyperalgesia	Area postrema (AP)
Ding et al. (2020)	Cystitis	Adult female Sprague Dawley rats	Pathological pain	Not mentioned
Han et al. (2020)	Cystitis	Adult female Sprague Dawley rats	Mechanical allodynia, depressive-like behaviors, and memory deficits	Hippocampus
Shin et al. (2021)	Atopic dermatitis	BALB/CJ mice (9-week-old pregnant female)	Autism	Not mentioned

## The Well-Studied Mechanism of Systematic Inflammation Induced Neuroinflammation: Immune-Related Factors

When it comes to neuroinflammation, immune-related factors and disruption of the BBB is widely being receipted as the most fundamental causes. The systematic inflammation leads to an increase in the circulating levels of pro-inflammatory cytokines such as IL-6, IL-18, TNF-α, and growth-related oncogene-α (GRO-α)/chemokine ligand 1(CXCL1) (Reichmann et al., 2015). Those cytokines enter CNS and trigger a series of subsequent inflammation reaction.

### Pathway for Cytokines to Enter Central Nerves System

**FIGURE 1:**
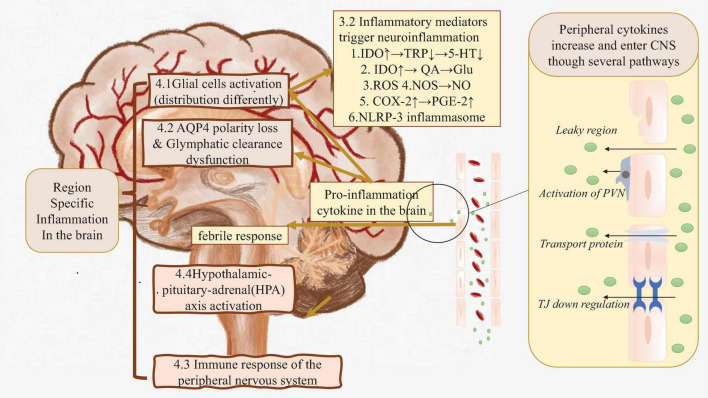
The real root of peripheral inflammation induced neuroinflammation. Right side: In the case of systematic inflammation, peripheral inflammation cytokines are produced. Those cytokines could flow into CNS directly or cause BBB leakage, finally cause pro-inflammation cytokine in the brain increasing. Left side: Those cytokines in CNS triggers inflammation by activating glial cells, causing glymphatic clearance dysfunction etc. Brown part: These factors may cause region-specific neuroinflammation. AQP4, Aquaporin 4; BBB, Blood-brain Barrier; C1q, Compliment 1q; CNS, Central nervous system; COX-2, Cyclooxygenase; Glu, Glutamate; HPA axis, Hypothalamic–pituitary–adrenal axis; IDO, Indoleaminepyrrole 2,3-dioxygenase; IL-1α, Interleukin-1α; NLRP-3, NACHT-LRR and pyrin (PYD) domain-containing protein-3; NO, Nitric oxide; NOS, Nitric oxide synthase; PGE-2, Prostaglandin E2; PNS, Peripheral nervous system; PSNS, parasympathetic nervous system; PVN, Perivascular macrophages; QA, Quinolinic acid; ROS, Reactive oxygen species; SNS, Sympathetic nervous system; TJ, Tight junction; TNF-α, Tumor necrosis factor-α; TRP, Tryptophan; 5-HT, 5-hydroxytryptamine.

Pro-inflammatory cytokines could enter the central nervures system by breaking down the BBB, or via several other pathways which will be discussed in detail later (Craig et al., 2022) (Figure 1 right side).

#### Circulating Inflammatory Mediators Induced Blood-Brain Barrier Leakage

The BBB consists of endothelial cells of the capillary wall, pericytes, and astrocytes. If any of the components of the BBB are missing, the barrier will collapse, and neuroinflammation will occur (Obermeier et al., 2013). Circulating inflammatory mediators (e.g., IL-6, TNF-α, and IL-1β) observed in systematic inflammation model animals may impede tight junction (TJ) regulation in brain endothelial cells, ultimately leading to a dysfunctional BBB marked by enhanced permeability (Banks, 2005; Zhou et al., 2006; McKim et al., 2018; Han et al., 2020). Also, previous studies demonstrated that microglia play a dual role in maintaining BBB integrity during sustained inflammation. Microglia phagocytose astrocytic end-feet and impair BBB function, leading to BBB leakage (Haruwaka et al., 2019). Further, vascular endothelial growth factor-A (VEGF-A) production in astrocytes is upregulated in response to IL-1β, inducing the endothelial nitric oxide synthase (eNOS)-dependent downregulation of tight-junction proteins claudin-5 (*Cldn5*) and occludin *(Ocln)* in endothelial cells, disrupting TJ and BBB integrity (Linnerbauer et al., 2020). BBB leakage accelerates a range of toxic circulating molecules, such as inflammatory cytokines, ions, and immune cells to access the brain microenvironment. These mediators further harm the BBB integrity (Uwe-Karsten Hanisch, 2007). The ROS and NO produced through cell respiration during inflammation also harms the BBB.

#### Other Pathways for Circulating Inflammatory Mediators to Enter Central Nerves System

Other pathways for circulating inflammatory mediators to enter CNS are: (i) through “leaky regions” in circumventricular organs which are found within the ependymal junctional region (Gotow and Hashimoto, 1982); (ii) through the utilization of endothelial transporter protein channels (Banks, 2005); (iii) via activating and inducing the release of local inflammatory mediators by endothelial cells and perivascular macrophages in the cerebral vasculature (Dantzer et al., 2008); (iv) via the activation and diapedesis of peripheral monocytes/macrophages and T-lymphocytes into the brain parenchyma (Shaftel et al., 2007).

### Inflammatory Mediators Trigger Neuroinflammation

Several animal studies have identified inflammatory markers, including IL-1, TNF-α, and IL-6 mRNA, in the hippocampal and cortical brain regions (Zonis et al., 2015; Heydarpour et al., 2016; Haj-Mirzaian et al., 2017; Han et al., 2018; He et al., 2021). After entering CNS, those inflammatory mediators also lead to additional cytokine production within the brain. These newly produced cytokines together with serological cytokines trigger neuroinflammation through following processes (Figure 1 left side).

#### Microglia Ctivation

Microglia are the resident immune cells in the CNS and occupy either a resting or activated state. Danger signals, including circulating inflammatory mediators, trigger resting microglia to transform to activated states (M1 and M2 phenotypes). M1 activated microglia produce pro-inflammatory mediators and are assumed to act as neurotoxic cells (Uwe-Karsten Hanisch, 2007; Hoogland et al., 2015), increasing indoleamine 2,3-dioxygenase (IDO), and inducing tryptophan [a precursor of serotonin (5-HT)] to a kynurenine pathway, leading to neuronal death (Douet et al., 2016). In addition, microglia interact with astrocytes during neuroinflammation. IL-1α, TNF-α, and complement 1q (C1q) secreted by microglia induce astrocytes to produce neurotoxic factors, decreasing phagocytic activity and expression of neurotrophic factors. These exacerbate level of neuroinflammation (Lull and Block, 2010; Linnerbauer et al., 2020). TNF-α also increases reactive oxygen species (ROS) secretion through cell respiration, leading to oxidative stress that aggravates inflammation.

#### Astroglia Activation

Astroglia play an essential role in neuroinflammation. Astrocytes respond to pro-inflammatory cytokines secreted by CNS-resident and CNS-recruited peripheral immune cells, thereby modulating the responses of neighboring cells throughout the CNS (Rothhammer and Quintana, 2015). Also, astrocytes respond to systematic inflammation through a COX2-dependent production of proteinoids. The coordinated down-regulation of COX-1 facilitates prostaglandin E2 (PGE2) production after Toll-like receptor 4 activation. These effects may increase cerebral blood flow responses to neuroinflammation (Font-Nieves et al., 2012). In systematic inflammation animal models, increased plasma cytokines are companied with the increased expression of Iba1 (a marker of activated microglia) and GFAP (a marker for astroglia) (Hoogland et al., 2015; Yang and Wang, 2015; Zonis et al., 2015). Nuclear translocation of NF-κB in astrocytes also elevates nitric oxide (NO) levels and accelerates inflammation progression through the nitric oxide pathway (Linnerbauer et al., 2020). Previous studies proved a significant increase in hippocampal TNF-α, iNOS expression, and nitrite content in colitis mice model (Heydarpour et al., 2016).

#### NACHT, LRR, and PYD Domain-Containing Protein 3 Inflammasome Activation

When peripheral organs get infected with bacteria, virus, and fugus, or exposure to environmental irritants, the peripheral inflammation or cellular stress will then activate NLRP3 inflammasome (Swanson et al., 2019). The NLRP3 inflammasome consists of a sensor (NLRP3), an adaptor (ASC) and an effector (caspase 1) (Swanson et al., 2019). During the inflammation process, the activated caspase 1 part of NLRP3 inflammasome in microglia and astrocytes triggers the maturation of cytokines including IL-1β, IL-18, etc. Furthermore, mature IL-1β binding to IL-1 receptors was reported to upregulate the expression of pro-IL-1β (Yazdi and Ghoreschi, 2016). Even though the detailed mechanism is still unclear, NLRP3 inflammasome is proved to associate with the pathogenesis of several inflammatory diseases including chronic colitis induced neuroinflammation and Cognitive impairment, and explains the different inflammation degree by age as reactive A1 astrocytes in the aging brain produces an exaggerated response to IL-1β (Pellegrini et al., 2020; He et al., 2021).

## Why is Peripheral Organs-Induced Neuroinflammation Limited to Specific Brain Regions?

In the case of neuroinflammation caused by inflammation of peripheral organs, brain regions are specifically affected. Even though previous studies have already proved several evidence that the fundamental mechanisms of peripheral inflammation-induced neuroinflammation is disruption of the BBB and immune-related factors (cytokine production, activation of glial cells, etc.), it fails to explain why neuroinflammation limits in special brain regions. To figure out the behind reason, some factors would be discussed as follow (Figure 1 left brown part).

### Immune-Related Factors: Distribution of Cytokines and Immune Cells

Cytokines are of crucial importance in the inflammation process. As discussed previously, cytokines originate not only from peripheral organs via the BBB, but are also locally produced in the CNS by glial cells (Albaret et al., 2020). Resultantly, the distribution of glial cells and region-dependent cytokine-production may be considered a potential explanation for region-specific neuroinflammation.

For cytokines, previous studies have shown that in the animal model of LPS induced systemic inflammation, the expression of IL-6, IL-1β, and cyclin-dependent kinase inhibitor p21Cip1 (p21) occurred in the hippocampus related to neuroinflammation caused by peripheral inflammation (Zonis et al., 2015). In the same animal model, p21 expression in the hippocampus contributes to restraining neuronal progenitor proliferation and protects these cells from inflammation-induced apoptosis (Zonis et al., 2013). Another study that examined mRNA expression of COX-2 (an enzyme related to production and release of PGE2) and GFAP (a marker for astrogliosis) in different brain regions (hypothalamus, amygdala, and hippocampus) concluded that IBD can lead to neuroinflammation in a bottom-up manner, and has region specific effects (Do and Woo, 2018). The higher expression of COX-2 (Font-Nieves et al., 2012) and GFAP (Yang and Wang, 2015) contributes to neuroinflammation.

For glial cells, astrocytes play a key role in neuroinflammation, and have a region-specific distribution of different subtypes in the brain (Christian Hochstim et al., 2008; Linnerbauer et al., 2020). A single-cell sequencing approach in adult mice tissues revealed that Astrocyte Sub Type 1 (AST1) was found at high levels in the subpial layer and hippocampus; AST2 and AST3, were found throughout cortex, AST4 was in subangular zone of hippocampus, and AST5 was in the subpial region, the stratum lacunosum-molecular and dentate gurus of hippocampus (Batiuk et al., 2020). Though expression of core genes are similar in different subtypes, still some differentially expressed genes are observed, which includs formation and/or maintence of the BBB (Mfsd2a), inmmune functions (Tril and Tlr3), glutamate uptake(Slc1a3), and other genes related to inflammation (Batiuk et al., 2020). Though details of the different functions of different astrocyte subtypes and differently distribution of astrocyte besides of cortex and hippocampus remain unclear, the region specific distribution of astrocyte could be an important explanation.

Also, generaly, microglia distributes in all brain regions and are 5 times more abundant in gray matter than in white matter. There are more microglia in the hippocampus, olfactory lobe and basal ganglia than in the thalamus and hypothalamus, and the least in the brainstem and cerebellum. Besides, inflammation progress is related to microglial remification or activation. Previous studies show that several factors such as chronic stress, and a combination of astrocyte-released cytokines, transforming growth factor-β (TGF-β), macrophage-colony stimulating factor (M-CSF) and granulocyte macrophage-colony stimulating factor (GM-CSF) is essential to microglial ramification (Walker et al., 2014). As a result, the different distribution of astrocyte would also induce region specific inflammation by influencing microglial ramfication.

### Glymphatic Clearance Disfunction

Glymphatic clearance was defined for the first time as a brain-wide anatomical pathway that facilitates the exchange of cerebrospinal fluid (CSF) and interstitial fluid (ISF), thereby promoting the clearance of various toxic waste products from the CNS (Iliff and Nedergaard, 2013). As a lymphatic-like system in the brain, glymphatic clearance drains protein waste into the cervical lymphatic system in humans and mice. This process depends on AQP4 channels expressed at high density on the astrocytic end feet abutting cerebral capillaries (Harrison et al., 2020). However, maturation of IL-1β, which binds to cognate receptors on astrocytes in response to active astrogliosis and produces inflammatory astrocyte (A1 astrocyte) which reduces AQP4 polarization and induces the dysfunction of glymphatic clearance (Iliff et al., 2014; Kress et al., 2014). As a result, an inflammatory environment would trigger glymphatic clearance dysfunction and lead the toxic products, cytokines or inflammation-related chemokines, to reach the CNS much more readily. Such state produces a vicious cycle that enhances neuroinflammation. A previous study also demonstrates that DSS treatment impaired glymphatic clearance and aggravated amyloid plaque accumulation and induced neuronal loss in the cortex and hippocampus (He et al., 2021). Furthermore, as the decreases of regional cerebral blood flow (rCBF) in the cerebral cortex and glucose uptake in all neocortical regions also observed in sepsis patients (Semmler et al., 2008). Even though in sepsis model, blood flow is influenced by blood pressure, heart rate and cardiac function, the CSF factor, besides of blood flow factors must be considered as an important factor that leads to region specific inflammation.

### The Immune Response of the Peripheral Nervous System

Immune cells express receptors for neuropeptides and neurotransmitters, and sense information from neurons, while neurons also accept stimuli from immune cells. Therefore, immune cells and neurons mutually influence each other in inflammatory diseases. This interaction in the peripheral immune system leads to inflammation in the brain via several pathways.

First, during peripheral inflammation, sensory neurons drive thermal and mechanical pain sensitivity and contribute to the febrile response which is considered a catalyst for neuroinflammation (Steinman, 2010; Udit et al., 2022). Some hypothesize of febrile response could be the pyrogenic cytokines IL-1, IL-6, and TNF-α get access to the hypothalamus through BBB or active transport mechanisms mediated by the cells surrounding the hypothalamus, then activate the febrile response indirectly by inducing local endothelial cells or microglial cells to secrete prostaglandin E2 (PGE2) to initiate a neuronal response that regulates the body temperature. Also, peripheral C5a stimulates PGE2 production in the liver and then trigger hypothalamus reaction by a neural pathway mediated by the vagus nerve and the nucleus tractus solitaries (Steinman, 2010). According to previous studies, several cytokines (e.g., IL-1β, IL-6, IL-10) are observed at elevated levels in the CNS and in peripheral circulation in heatstroke models (Leon et al., 2006; Biedenkapp and Leon, 2013), which gives evidences that febrile response leads to a systematic inflammation status and also relates with neuroinflammation.

Second, since sensory neurons are afferents, excessive sensory stimulation may cause overactivation and cytokine production. A previous study employed a chronic constriction nerve injury pain model to demonstrate the interactive role of presynaptic sensitivity to TNF-α and the adrenergic auto receptor. This study suggested that an important role that TNF-α plays in adrenergic neuroplastic changes in a region of the brain such as locus coeruleus and hippocampus, and implicated in the pathogenesis of neuropathic pain (Covey et al., 2000). However, the same peripheral nervous system (PNS) pathway can exert differential and even opposing effects on immune cells depending on the specific disease, tissue, and timing contexts. As neurons with the same or similar functions are grouped together, one mechanism that is likely to underlie this heterogeneous response is the presence of distinct subsets of neurons within the various PNS pathways (Udit et al., 2022).

### The Hypothalamic–Pituitary–Adrenal Axis Activation

Stress is a crucial factor during chronic visceral inflammation (Furutama et al., 2020). Activation of peripheral vagal nerve afferents that relay cytokine signals to the nucleus of the solitary tract and hypothalamus are referred to as the HPA axis (Ghia et al., 2006; Chavan et al., 2017). Activated HPA axis regulates the neuroinflammation condition in an interactional way (O’Callaghan and Miller, 2019). Acute and chronic stress activate the HPA axis to produce a variety of stress hormones (Sudo, 2014). Binding to receptors on microglia, corticotropin-releasing hormone (CRH) activates microglia. adrenocorticotropic hormone (ACTH) can inhibit the activity of immune cells and produce anti-inflammatory effects through the central melanocortin receptor. Glucocorticoids (GC) and mineralocorticoid (MC) can bind to glucocorticoid receptors (GR) and mineralocorticoid receptors (MR), thereby playing an important role in the central inflammatory response. Depending on the distribution of these receptors, inflammation is triggered differently in different brain regions. For instance, mineralocorticoid receptor (MR) is prevalent in the hippocampus, amygdala, and paraventricular nucleus of hypothalamus (PVN) neurons – potentially leaving them more susceptible to stress induced neuroinflammation. Also, the stress process is associated with brain region-dependent alterations of neuropeptide Y (NPY), NPY receptor Y1, CRH, CRH receptor 1, BDNF, and glucocorticoid receptor expression (Herman et al., 2012; Reichmann et al., 2015). BDNF is a factor related to neurogenesis, neural plasticity, and activation of astrocytes and microglia (Ding et al., 2020), has been proved to have a suppression expression pattern in hippocampus, prefrontal cortex and striatum response to stress depending on the brain region (Bath et al., 2013). As BDNF is mainly expressed in the cortex and hippocampus, inflammation may easily affect these regions.

## Discussion

According to previous animal and clinical studies, neuroinflammation may be caused by inflammation of several peripheral organs such as colitis, PD, gastritis, pancreatitis, arthritis, cystitis, atopic dermatitis, PBC, CFS/ME, and COPD (Lin et al., 2018; Albaret et al., 2020; Ding et al., 2020; Furutama et al., 2020; Matsushita et al., 2021; Shin et al., 2021). Given the neuropsychiatric symptoms and the results of biochemical experiments, research has identified that the hippocampus, cortex, amygdala, and hypothalamus are most affected by peripheral-induced neuroinflammation (Reichmann et al., 2015; Riazi et al., 2015; Zonis et al., 2015; Do and Woo, 2018; Han et al., 2018; Peppas et al., 2021).

Besides the mechanism we have discussed before, other factors including gut-brain axis (Pellegrini et al., 2020; Peppas et al., 2021), joint-brain axis (Suss et al., 2020), and liver-brain axis (Matsubara et al., 2022), as well as a multiorgan network also lead to neuroinflammation. Additionally, hypoxia has been shown to worsen brain inflammation in DSS-induced colitis models and induce the development of cerebral edema in mice at high altitudes by activating microglia, producing pro-inflammatory mediators and down-regulating TJs (Han et al., 2020). Neuroinflammation in COPD patients is also related to hypoxia (Pelgrim et al., 2019).

Though the most basic trigger of neuroinflammation is immune-related factors including leakage of the BBB, there is no evidence that the distribution of BBB has region specificity. However other factors trigger neuroinflammation such as region dependent crucial gene expression (cytokines, receptors, and other proteins) (Reichmann et al., 2015), dysfunction of glymphatic clearance (Iliff and Nedergaard, 2013; Iliff et al., 2014), PNS regulation (Udit et al., 2022), febrile response (Steinman, 2010; Udit et al., 2022) could give explanation to the region specificity of neuroinflammation. Especially, diversity of neuronal and glial cells in different regions may be considered a possible explanation for regionalized neuroinflammation.

We have already discussed the region-specific distribution of astrocyte before. As for neuron diversity, according to previous research, mammalian brain cells show remarkable diversity in gene expression, anatomy, and function. A previous study has described the DNA methylation patterns of excitatory neurons in the cortex and hippocampus, which both varied continuously across spatial gradients (Liu et al., 2021). Another study has identified the single-cell morphological diversity of neurons, which revealed 11 major projection neuron types with distinct morphological features and transcriptomic identities, though the specific distribution and functional differences of different types of neurons are unclear (Peng et al., 2021).

## Strengths and Limitations

### Strengths

In clinical practice, it is difficult to treat peripheral inflammatory disease because of the neuroinflammation caused by peripheral inflammation. A range of psychological symptoms such as depression, anxiety, and cognitive dysfunction induced by neuroinflammation can not only physically afflict the It is very important to find an effective treatment for neuroinflammation to completely cure peripheral inflammation. However, it is very difficult to find an efficient treatment for each systemic inflammatory disease. We commented on the mechanisms by which peripheral inflammation induces neuroinflammation and the causes of region-specific neuroinflammation. In particular, region-specific neuroinflammation can occur in any inflammatory disease, so elucidation of region-specificity will lead to smooth treatment and complete cure of various inflammatory diseases. Our review may provide guidance for further research on neuroinflammation and the development of new neuroinflammation therapies.

### Limitations

Although much research on peripheral inflammation-induced neuroinflammation has been done in recent years, there are some unresolved limitations. First, the assessment of inflammation in previous studies is different for each disease. Since there are various evaluations such as increased expression of inflammation-promoting factors, increased inflammatory cytokines, activation of microglia and disruption of the blood-brain barrier, it is difficult to compare the degree of neuroinflammation between diseases. Second, no studies have been found that analyze neuroinflammation in all brain regions. The neuroinflammation vulnerability in the area remains unknown because there are no results. Third, the degree of systemic inflammation induced by peripheral inflammation varies depend on research, so mutual comparison may not make sense. In peripheral inflammatory diseases, the degree of neuroinflammation and the area of inflammation may change between severe and mild, acute and chronic phases. Third, the degree of systemic inflammation due to peripheral inflammation varies from study control to control, so mutual comparison may not be meaningful. In peripheral inflammatory diseases, the degree of neuroinflammation and the area of inflammation may change between severe and mild, acute and chronic phases. To elucidate the mechanism of systemic inflammation-induced neuroinflammation, it is necessary to conduct morphological studies with the same inflammatory criteria in various peripheral inflammatory diseases (colitis, pneumonia, hepatitis, dermatitis, etc.).

## Author Contributions

YS and YK designed the review, gathered the previous articles, and wrote the manuscript. SS supervised this review and provided intellectual directions. All authors discussed and commented on this manuscript.

## Conflict of Interest

The authors declare that the research was conducted in the absence of any commercial or financial relationships that could be construed as a potential conflict of interest.

## Publisher’s Note

All claims expressed in this article are solely those of the authors and do not necessarily represent those of their affiliated organizations, or those of the publisher, the editors and the reviewers. Any product that may be evaluated in this article, or claim that may be made by its manufacturer, is not guaranteed or endorsed by the publisher.
